# Allergen-specific sublingual immunotherapy altered gut microbiota in patients with allergic rhinitis

**DOI:** 10.3389/fcimb.2024.1454333

**Published:** 2024-11-08

**Authors:** Jing Wu, Dan Wang, Wen-Jun He, Jun-Yang Li, Xi Mo, You-Jin Li

**Affiliations:** ^1^ Department of Otorhinolaryngology, Hainan Branch, Shanghai Children's Medical Center, School of Medicine, Shanghai Jiao Tong University, Sanya, China; ^2^ Central Lab, Hainan Branch, Shanghai Children's Medical Center, School of Medicine, Shanghai Jiao Tong University, Sanya, China; ^3^ Pediatric Translational Medicine Institute, Shanghai Children's Medical Center, School of Medicine, Shanghai Jiao Tong University, Shanghai, China; ^4^ Department of Science and Education, Hainan Branch, Shanghai Children's Medical Center, School of Medicine, Shanghai Jiao Tong University, Sanya, China; ^5^ Department of Otolaryngology, Shanghai Children’s Medical Center, School of Medicine, Shanghai Jiao Tong University, Shanghai, China

**Keywords:** allergic rhinitis, dermatophagoides farinae, allergen-specific sublingual immunotherapy, gut microbiota, Streptococcus parasanguinis_B

## Abstract

**Introduction:**

Allergen-specific immunotherapy (AIT) induces long-term immune tolerance to allergens and is effective for treating allergic rhinitis (AR). However, the impact of sublingual immunotherapy (SLIT) on gut microbiota from AR patients and its correlation with treatment efficacy remains unclear.

**Methods:**

In the present study, we enrolled 24 AR patients sensitized to Dermatophagoides farinae (Der-f) and 6 healthy donors (HD). All AR patients received SLIT treatment using standardized Der-f drops. Stool samples were collected from AR patients before treatment, and 1- and 3-months post-treatment, as well as from HD, for metagenomic sequencing analysis.

**Results:**

AR patients had significantly lower richness and diversity in gut microbiota compared to HD, with notable alterations in composition and function. Besides, three months post-SLIT treatment, significant changes in gut microbiota composition at the genus and species levels were observed in AR patients. *Streptococcus parasanguinis_B* and *Streptococcus parasanguinis*, which were significantly lower in AR patients compared to HD, increased notably after three months of treatment. LEfSe analysis identified these species as markers distinguishing HD from AR patients and AR patients pre- from post-SLIT treatment. Furthermore, changes in the relative abundance of *S. parasanguinis_B* were negatively correlated with changes in VAS scores but positively correlated with changes in RCAT scores, suggesting a positive correlation with effective SLIT treatment.

**Discussion:**

SLIT treatment significantly alters the gut microbiota of AR patients, with *S. parasanguinis_B* potentially linked to its effectiveness. This study offers insights into SLIT mechanisms and suggests that specific strains may serve as biomarkers for predicting SLIT efficacy and as modulators for improving SLIT efficacy.

## 1 Introduction

Allergic rhinitis (AR) is a chronic inflammatory disease affecting the nasal mucosa, characterized by nasal congestion, rhinorrhea, postnasal drainage, sneezing, and itching of the eyes, nose, and throat (Bernstein et al., 2024). AR impacts a significant portion of the global population, with incidence rates ranging from 5% to 50% across diverse countries and regions (Strachan et al., 2022; Wise et al., 2023). It is notable that the incidence of AR has been on the rise globally, particularly in the industrialized world, thereby exerting a considerable impact on global health (Schwindt and Settipane, 2012). The burden of AR in children is particularly significant as it can profoundly affect their quality of life, emotional well-being, academic performance, and social and cognitive functioning (Meltzer et al., 2009). Additionally, uncontrolled AR during childhood is linked to an elevated risk of developing other atopic diseases, such as asthma and allergic conjunctivitis (Licari et al., 2023).

The management of AR includes allergen avoidance, pharmacotherapy (oral and/or nasal corticosteroids and/or antihistamines), and Allergen-specific-immunotherapy (AIT) (Hossenbaccus et al., 2020). AIT is recommended for AR patients who are sensitive to airborne allergens and experience persistent symptoms despite receiving optimal medication and interventions to reduce environmental exposure. Among the available routes of allergen immunotherapy, subcutaneous and sublingual administration have been proven to be the most effective (Bernstein et al., 2024). AIT promotes allergen tolerance through various mechanisms, including reducing local mast cells, basophils, eosinophils, and type 2 innate lymphoid cells by modulating the innate immune system. Additionally, it may impact the adaptive immune system by inducing allergen-specific IgG blocking antibodies, immunosuppressive cytokines, regulatory T cells, and B cells (Alvaro-Lozano et al., 2020; Drazdauskaitė et al., 2020). However, the efficacy of AIT in treating AR varies among individual patients, emphasizing the need for further research to explore the mechanisms influencing its effectiveness.

Numerous studies have well demonstrated the involvement of gut microbiota in the pathogenesis of AR (Kaczynska et al., 2022). AR patients exhibit altered diversity, composition, and functionality of gut microbiota compared to healthy controls (Zhou et al., 2021; Wan et al., 2023). AIT has been demonstrated to induce changes in the gut microbiota of patients with various atopic diseases, correlating with treatment outcomes (He et al., 2021; Liu et al., 2023). For instance, a study on atopic dermatitis (AD) revealed alterations in the composition and function of the gut microbiota in AD patients following AIT treatment. The abundance of *Brevundimonas vesicularis* in the gut of AD patients was positively correlated with disease severity and decreased following AIT (Liu et al., 2023). However, the changes in gut microbiota during AIT treatment for AR, as well as their role in AIT efficacy, remain unclear to date.

In this study, we compared the differences in gut microbiota between healthy individuals and children with AR sensitized to Dermatophagoides farinae (Der-f), as well as the variations in gut microbiota among AR patients before sublingual immunotherapy (SLIT) with Der-f drops and at 1 month and 3 months post-treatment. We aim to investigate whether SLIT alters the composition or function of the gut microbiota and whether these changes correlate with the patient’s response to therapy. Moreover, we are endeavoring to propose potential optimization strategies to enhance AIT efficacy by modulating the gut microbiota, thereby improving the treatment outcomes of AR.

## 2 Material and methods

### 2.1 Study participants

Twenty-nine pediatric AR patients, who received SLIT treatment with Der-f drops at the Department of Otorhinolaryngology at Shanghai Children’s Medical Center from august 2022 to august 2023, were enrolled in this study. The diagnosis of AR was based on the 2022 updated Chinese guideline for the diagnosis and treatment of pediatric allergic rhinitis (Bousfiha et al., 2022). Inclusion criteria for AR patients were as follows: (i) aged between 5 and 12 years old; (ii) sensitized to Der-f, confirmed by serum levels of Der-f specific IgE greater than grade 3; (iii) no history of AIT treatment; (iv) meeting the valid indications for AIT (Lee et al., 2023); (v) voluntarily participating in the study and providing stool samples; (vi) no other allergic diseases, such as asthma, atopic dermatitis. Healthy pediatric donors were included if they met the following criteria: (i) no history of allergic conditions; (ii) absence of acute infections; and (iii) voluntary participation in the study; (iv) no family history of allergic disease. Exclusion criteria for both groups were: (i) recent use of antibiotics, prebiotics, probiotics, or synbiotics within the past month; (ii) severe gastrointestinal abnormalities; and (iii) any other severe diseases, such as malignancies, autoimmune diseases, or immunodeficiencies. AR Patients were given SLIT treatment by using standardized allergen Der-f drops (Chanllergen; Zhejiang Wolwo Bio-Pharmaceutical Co., Ltd., Zhejiang, China) according to the product instructions. Stool samples from 5 AR patients did not pass the quality test and were subsequently excluded, resulting a total of 24 AR patients included in this study. The flowchart of the present study was shown in **Supplementary Figure S1**.

The study was approved by the Institutional Review Board and the Ethics Committee of Shanghai Children’s Medical Center (SCMCIRB-YPDWJW2022001), and written informed consents were obtained from all donors and/or their parents.

### 2.2 Total serum and allergen-specific serum IgE levels measurement in children

The serum samples were obtained from the enrolled children for total IgE levels and allergen-specific IgE levels. This testing was conducted using the ImmunoCAP 1000 system from ThermoFisher Scientific, Uppsala, Sweden. The specific IgE concentrations were measured for the seven common aeroallergens found in Shanghai, which comprise Der-f and Dermatophagoides pteronyssinus (Der-p), animal hair (cat hair and dog hair), mold, weed pollen, and tree pollen. The results were automatically calculated based on the fluorescence responses. For total IgE levels, a positive value was defined as exceeding 60 IU/mL in the pediatric population. The levels of specific IgE (sIgE) were classified into six grades (0.35-0.70 kU/L (class 1), 0.7-3.5 kU/L (class 2), 3.5-17.0 kU/L (class 3), 17.0-50.0 kU/L (class 4), 50.0-100.0 kU/L (class 5), and >100.0 kU/L (class 6)). Values below 0.35 kU/L indicated the subject as non-sensitized.

### 2.3 Sample collection and processing

Fecal samples from all participants were collected in sterile plastic tubes, transported to the laboratory in ice-filled coolers within 2 hours of collection, and subsequently stored at −80°C prior to analysis. Fecal samples were collected at baseline (before SLIT treatment), 1 month post-treatment, and 3 months post-treatment.

### 2.4 Metagenomic shotgun sequencing

The total genomic DNA isolated from fecal samples was fragmented to produce fragments averaging approximately 400 base pairs in length (ranging from 200 to 600 bp). Subsequently, the libraries were constructed using the NEBNext^®^ UltraTM DNA Libraries prep kit for Illumina (NEB, USA), and subjected to 2 × 150 bp paired-end metagenomic shotgun sequencing using the NovaSeq 6000 platform at Shanghai GeneSky Biological Technology Co., Ltd. (China).

Raw reads were filtered by using fastp v0.19.5 software. Human genome sequences were then removed by using Bowtie 2. FastQC v0.11.8 was used to check the quality of the reads. The clean reads of each sample were then assembled using Megahit v.1.2.9, selecting contigs with a length of ≥200 bp as the assembly output. Redundant contigs were then removed using CD-HIT v4.8.1. Subsequently, MetaGeneMark v3.38 was utilized to annotate the assembled contigs. High-quality target genes, each with a length of ≥100 bp, were clustered using CD-HIT v4.8.1 to produce a nonredundant set. Binning was performed with metaBAT2 v2.12.1 using default parameters. Annotation of gene sets for archaea, bacteria, eukaryota, viroids, and viruses was conducted using Diamond (v0.9.36) by using the National Center for Biotechnology Information (NCBI) and Genome Taxonomy (GTDB) database.

### 2.5 Evaluation of clinical efficiency of SLIT treatment

Treatment effectiveness was assessed by examining changes in both the Visual Analog Scale (VAS) score and the Rhinitis Control Assessment Test (RCAT) score following one year of treatment. The VAS score utilizes a scale from 0 to 10, where patients rate the severity of symptoms such as nasal congestion, sneezing, itching, and rhinorrhea. A score of 0 indicates no symptoms, while 10 signifies the most severe symptoms imaginable. Patients indicate their discomfort level on this scale, offering a subjective measure of symptom severity. On the other hand, the RCAT is a questionnaire-based assessment designed to gauge rhinitis symptom control. It consists of six items covering nasal congestion, sneezing, watery eyes, sleep disturbance, activity avoidance, and self-assessed control. RCAT scores range from 6 to 30, with higher scores indicating better control of rhinitis symptoms. Treatment groups were classified as effective when there was improvement in either the VAS score or the RCAT score (E-SLIT, effective subcutaneous immunotherapy). Conversely, treatment groups were classified as ineffective when there was no improvement in either the VAS score or the RCAT score (I-SLIT, ineffective subcutaneous immunotherapy).

### 2.6 Statistical analysis

The α-diversity and β-diversity analyses of the gut microbiota were conducted and visually depicted using the vegan package (v2.5.6) and fossil packages in R (v0.3.7). Differences in the relative abundance of taxa between groups were assessed using the non-parametric Wilcoxon rank-sum test. For the identification of differentially abundant bacterial taxa between groups, linear discriminant analysis effect size (LEfSe) analysis was performed. Bacterial taxa with a linear discriminant analysis (LDA) score > 2 and p < 0.05 were deemed statistically significant. Kyoto Encyclopedia of Genes and Genomes (KEGG) and COG (Clusters of Orthologous Group) functions analysis were carried out using PICRUSt analysis against the database. STAMP software was utilized to confirm statistical differences (*p* < 0.05). Exploration of microbial markers for AR patients was conducted using a random forest model, with mean decrease Gini used for variable ordering and selection.

All parametric clinical data underwent analysis using SPSS22.0 software. The normality of variables was assessed using the Shapiro−Wilk test. Quantitative variables meeting the criteria of normality and homogeneity of variance were expressed as mean and standard deviation (mean ± SD), while those failing to meet these criteria were described as median and interquartile difference (IQR). Quantitative variables were analyzed using Student’s t-test, Mann minus;Whitney test, or Wilcoxon test, as appropriate. Categorical variables were tested using Chi-square or Fisher’s exact test. *p* value < 0.05 was considered to be statistically significant.

## 3 Results

### 3.1 Demographics and clinical characteristics of the participants

A total of 30 pediatric participants, including 24 AR patients and 6 healthy donors (HD), were included in the present study. The demographics and clinical characteristics of the study participants are listed in **Table 1**. There were no significant differences in age, gender, Body Mass Index (BMI), mode of delivery, family history of allergy, feeding patterns before 6 months of age, and defecation between the two groups. All patients with AR were sensitized to both Der f and Der p. Among these patients, eight children also reacted to additional minor allergens, including animal hair and molds.

**Table 1:** Demographics and clinical characteristics of study participants.

Characteristics	HD	AR	*P* value
Subjects (n)	6	24	
Age (years, Mean ± SD)	6.43 ± 4.45	8.58 ± 2.59	0.07
Gender, n (%)			
Male	5 (83.3%)	17 (70.8%)	1.000 ^a^
Female	1 (16.7%)	7 (29.2%)	
BMI (kg/m^2^)	19.33 ± 4.85	17.13 ± 3.68	0.162
Mode of delivery, n (%)			
Vaginal	1 (16.7%)	12 (50.0%)	0.196 ^a^
Cesarean	5 (83.3%)	12 (50.0%)	
Family history of allergy, n (%)			
No	5 (83.3%)	11 (45.8%)	0.175 ^a^
Yes	1 (16.7%)	13 (54.2%)	
Feeding patterns before 6 months of age, n (%)			
Exclusively breastfeeding	6 (100.0%)	12 (50.0%)	0.116 ^a^
Mixed feeding	0 (0.0%)	10 (41.7%)	
Formula feeding	0 (0.0%)	2 (8.3%)	
Defecation, n (%)			
Regular defecation	5 (83.3%)	12 (50.0%)	0.196 ^a^
Constipation	1 (16.7%)	12 (50.0%)	
Total IgE	78.28 ± 84.37	622.47 ± 605.08	<0.001
Der-f sIgE			
III		3	
IV		6	
V		9	
VI		6	
Der-p sIgE			
III		1	
IV		4	
V		12	
VI		7	

HD, Healthy donor; AR, Allergic rhinitis; BMI, Body Mass Index; Der-f, Dermatophagoides farina; Der-p, Dermatophagoides pteronyssinus; sIgE, specific IgE.

a Fisher’s exact test.

### 3.2 AR patients exhibit altered gut microbial diversity and composition

**Figure 1:**
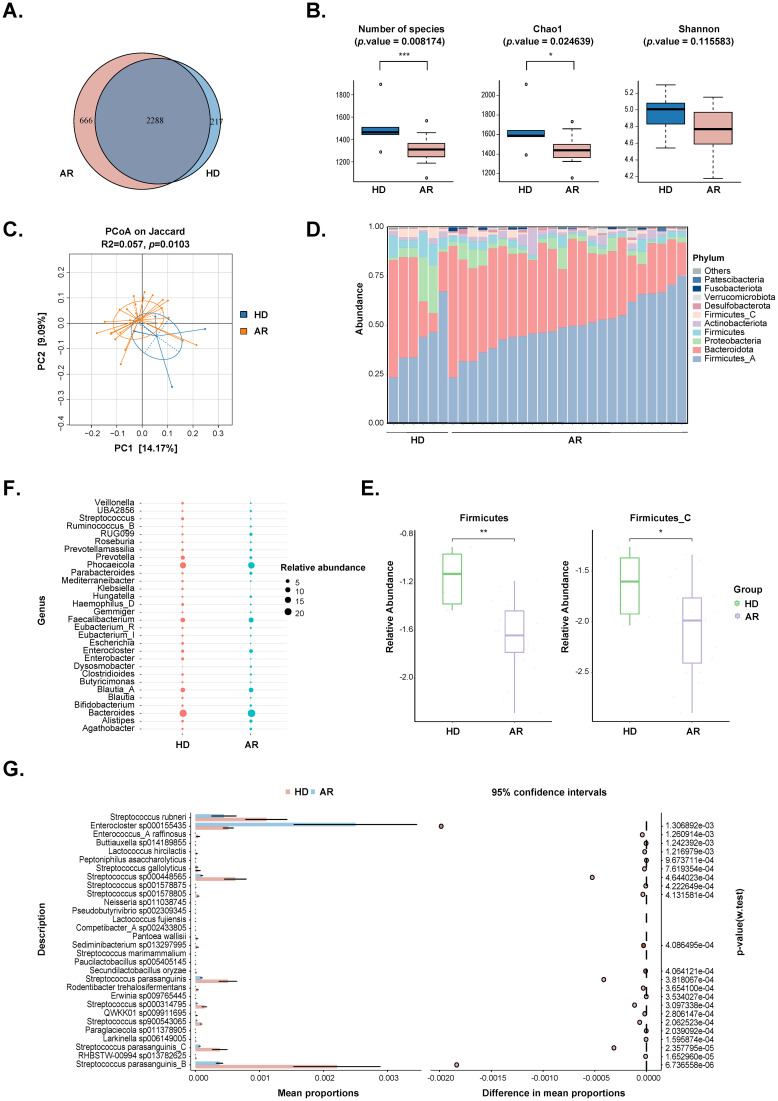
Comparisons of the gut microbiota diversity and abundance between AR patients and healthy donors (HD). **(A)** Venn diagram of the observed ASVs in AR patients and HD. **(B)** *Alpha* diversity comparison including Number of species, Chao1 and Shannon index. **(C)** Principal coordinate analysis based on Jaccard distances was shown along the first two principal coordinate (PC) axes. Each point represented a single sample. **(D)** Bar plots showing the relative abundance of microbiota at phylum level of individuals from the two groups, with different colors corresponding to different phyla. **(E)** The relative abundance of *Firmicutes* and *Firmicutes_C* were shown between the two groups. **(F)** Bubble plot of the relative abundance of microbiota at genus level between the two groups. Only the 30 most abundant taxa in all samples were represented. Bubble size represented the abundance of gut microbiota. **(G)** STAMP analysis of the relative abundance of microbiota at species level between the two groups. Only the 30 most abundant taxa in all samples were represented. *, p value<0.05; **, p value<0.01; ***, p value<0.005.

Fecal metagenomic sequencing was used to compare the gut microbiome composition and function between AR patients and HD. The results revealed that a total of 2288 amplicon sequence variants (ASVs) were shared between the two groups, with 666 ASVs unique to the AR group and 217 ASVs unique to the HD group (**Figure 1A**). Alpha diversity, assessed by the observed number of ASVs, Chao1 index, Shannon index, and Simpson index, demonstrated that the bacterial gut microbiota of AR patients exhibited significantly lower richness compared to that of HD (*P*=0.008174 for observed number of ASVs and *P*=0.024639 for Chao1 index), while the evenness of the bacterial gut microbiota was comparable between the two groups (**Figure 1B**; **Supplementary Figure S2A**). Moreover, β-diversity was evaluated by principal coordinates analysis (PCoA) based on Bray-Curtis distance, which is sensitive to differences in relative abundance for the most abundant ASVs, as well as binary Jaccard distance, which only accounts for the presence/absence of operational units and is thus more sensitive to gut-microbiota changes driven by rare ASVs. The results indicated that, although there was no a separation between the two groups through PCoA based on Bray-Curtis distance (**Supplementary Figure S2B**), there was a separation between the two groups through PCoA based on binary Jaccard distance (*P*=0.0103; **Figure 1C**). These findings indicate that the richness and diversity of the gut microbiota in AR patients are significantly different from those in HD.

Differential microbes were observed from phylum to species levels (**Figures 1D-G**; **Supplementary Table S1**). The top 10 phyla in the relative abundance of gut microbiota were shown in **Figure 1D**, with *Firmicutes* and *Firmicutes_C* significantly decreasing in the AR group (**Figure 1E**). At the genus levels, 110 genera were identified to be significantly different between the two groups. The top 30 genera were displayed in **Figure 1F**, and *Streptococcus* and *Escherichia* were significantly decreased, while *Parabacteroides* and *Dysosmobacter* were significantly increased in AR group. At the species levels, a total of 328 species were significantly different between the two groups. The top 30 species were shown in **Figure 1G**, and the strain exhibiting the most significant difference beening *Streptococcus parasanguinis_B*.

**Figure 2:**
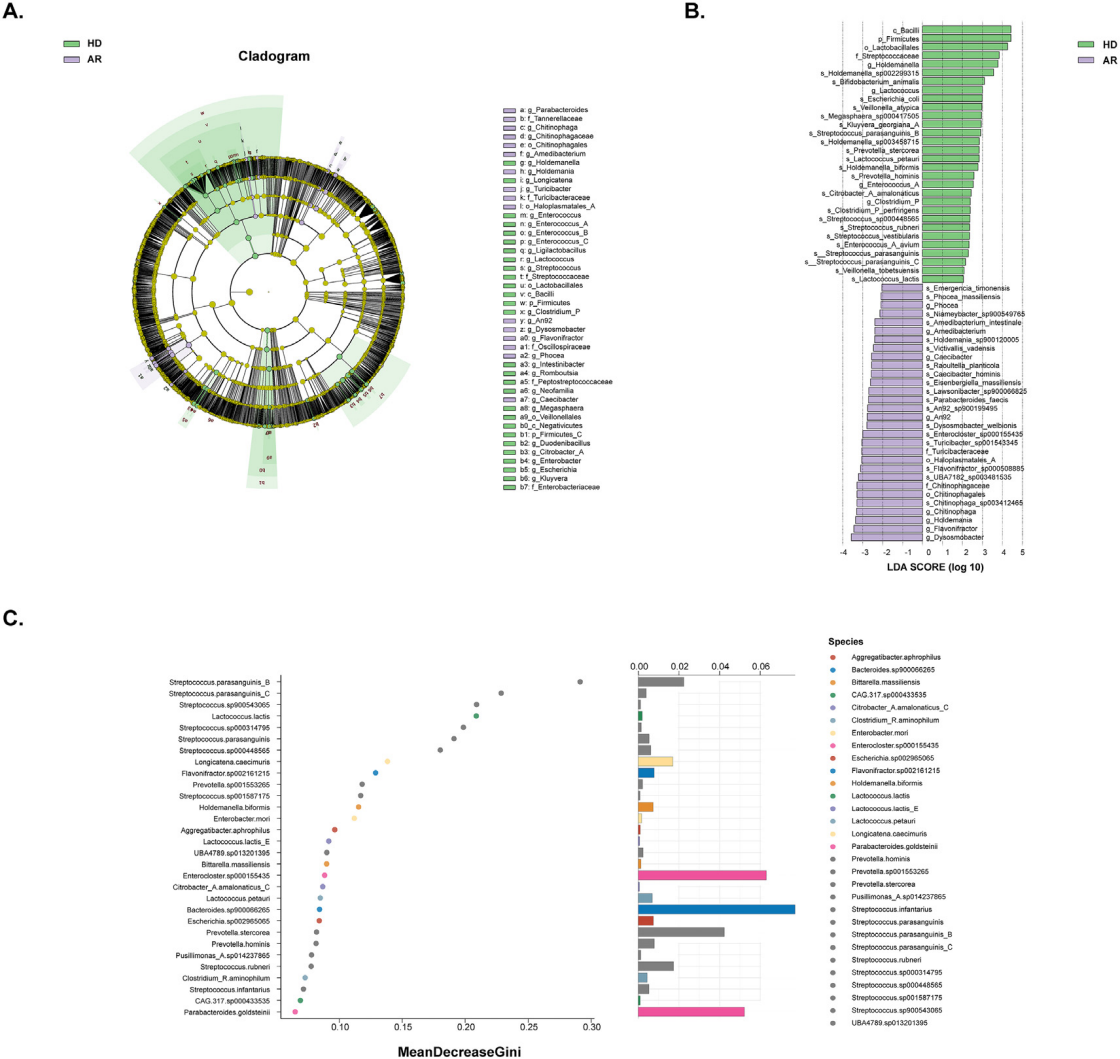
Analysis of significantly different gut microbiota composition between AR patients and healthy donors (HD) by using LEfSe analysis and random forest analysis. **(A)** Different structures of gut microbiota in AR patients and HD by LEfSe analysis. A cladogram plot illustrated the distinct species from phylum to genus levels, with node size indicating taxa abundance. **(B)** A histogram displayed the linear discriminant analysis (LDA) scores for key bacteria classifications with varying abundances between AR patients and HD. Significance was determined using LDA effect size (LEfSe) with P<0.05 (Wilcoxon test) and LDA score>2. The top 30 taxa with the most significant differences in each group were shown. **(C)** A random forest plot showed the 30 most predictive bacterial taxa distinguishing AR patients from HD.

Linear discriminant analysis effect size (LEfSe) was used to employ the significant differences in microbial taxa between the two groups (bacterial biomarkers), and 122 discriminative features were identified as having differential relative abundance between the two groups (LDA score>2, P<0.05; **Supplementary Table S2**). The top 30 microbial taxa with most significant differences in both groups were shown in **Figures 2A, B**. Furthermore, random forest ranked the top 30 species that contribute to the discrimination of AR and HD groups (**Figure 2C**). By integrating the results of LEfSe and random forest, 10 species of bacteria were identified. Among them, *S. parasanguinis_B*, *S. parasanguinis_C*, *Lactococcus.lactis*, *S. parasanguinis*, *S. sp000448565*, *Holdemanella biformis*, *Lactococcus petauri*, *Prevotella stercorea*, *and S. rubneri* were significantly enriched in the HD group, while *Enterocloster.sp000155435* exhibited higher abundances in the AR group.

### 3.3 Functional profile of the gut microbiota between AR patients and HD

**Figure 3:**
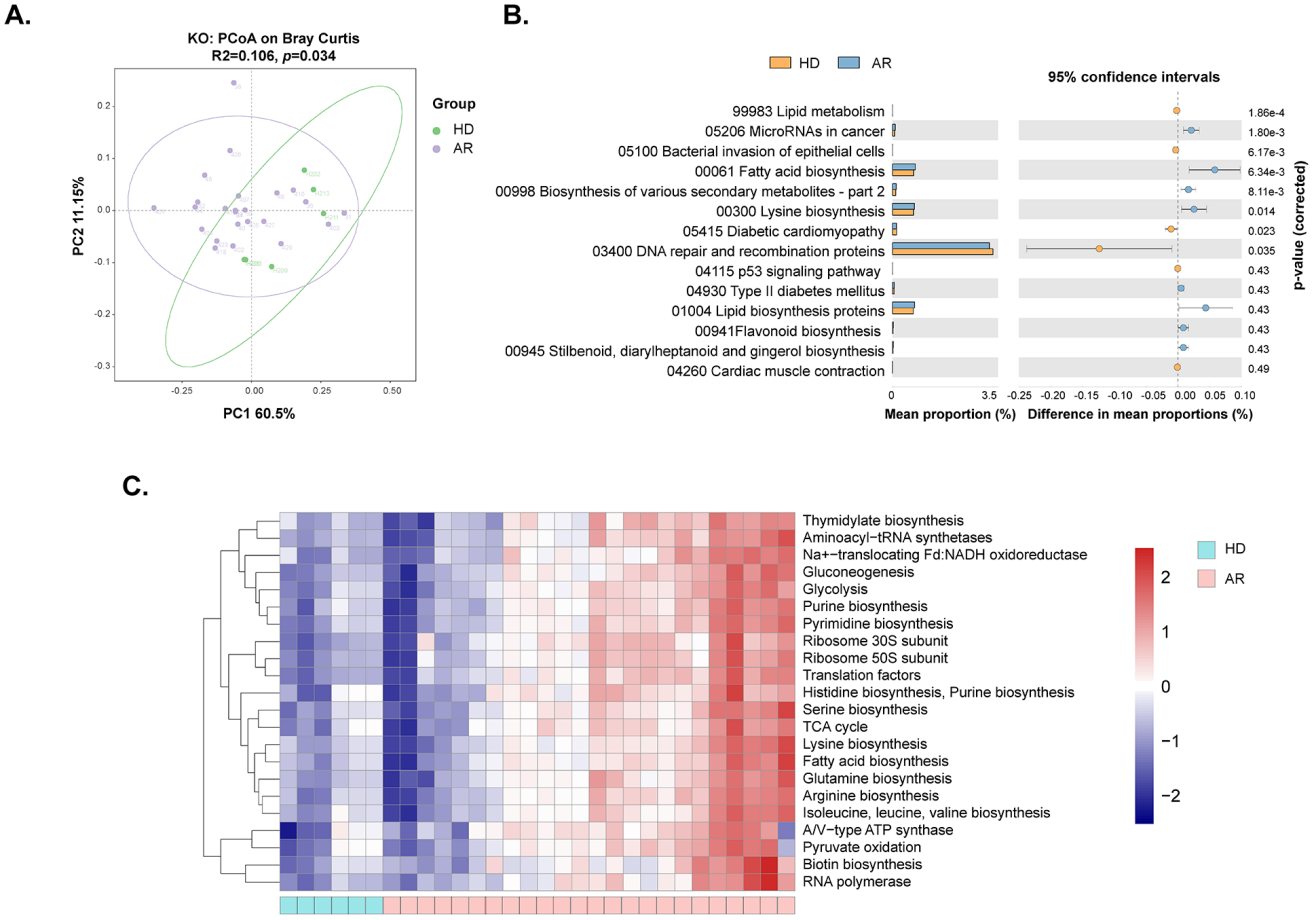
Functional analysis of gut microbiota between AR patients and healthy donors (HD). The genes underwent functional annotation using the Kyoto Encyclopedia of Genes and Genomes (KEGG) database and Cluster of Orthologous Groups (COG) through PICRUSt analysis. **(A)** Principal coordinate analysis (PCoA) of KEGG orthology (KO) based on Bray-Curtis distance metrics between AR patients and HD. **(B)** STAMP analysis identified 14 KEGG pathways significantly differing in abundance between AR patients and HD. **(C)** Heat map showing 22 predicted COG functional pathways significantly distinct between AR patients and HD.

To further understand the changes in gut microbial function and metabolic activity between AR patients and HD, KEGG and COG analyses were conducted in the present study. The dimensionality reduction PCoA revealed a significant difference in the KEGG orthology (KO) between the two groups (**Figure 3A**). Further analysis indicated that many metabolism-related pathways, including Gluconeogenesis/Glycolysis, Purine/Pyrimidine metabolism, Amino acid (Histidine/Serine/Lysine/Glutamine/Arginine/Isoleucine/Leucine/Valine) biosynthesis, Fatty acid metabolism, TCA cycle, etc., were enriched in AR patients (**Figures 3B, C**).

### 3.4 Demographics and clinical characteristics of the AR patients treated with subcutaneous immunotherapy (SLIT)

All 24 AR patient received SLIT treatment, and the treatment efficiency was evaluated by analyzing changes of the VAS score and RCAT score. Six patients were lost to follow-up, and VAS and RCAT scores were obtained from 18 patients. Individual scores are provided in **Supplementary Table S3**. It was observed that both VAS and RCAT scores significantly improved in patients after SLIT (*P*=0.004 and 0.009, respectively). Among the sub-items of RCAT, nasal congestion, sneezing, and self-assessed control also demonstrated significant improvement (*P*=0.022, 0.038, and 0.022, respectively; **Table 2**).

**Table 2:** Comparison of VAS score and RCAT score before and after subcutaneous immunotherapy treatment in AR patients.

Characteristics	Pre-SCIT Median (IQR)	Post-SCIT Median (IQR)	*P* value
VAS score	7 (6, 8)	5 (2, 6.25)	0.004
RCAT score	21 (12, 27)	25.5 (22.5, 27)	0.009
Nasal congestion	2.5 (2, 3.25)	4 (3, 4.25)	0.022
Sneezing	3 (2, 4.25)	4 (3, 5)	0.038
Watery eyes	4 (2, 5)	5 (3.75, 5)	0.226
Sleep interference	4 (2.75, 5)	5 (3, 5)	0.090
Activity avoidance	5 (4, 5)	5 (5, 5)	0.117
Self-assessed control	3 (2.75, 4)	4 (3, 4)	0.022

VAS, Visual Analog Scale; RCAT, Rhinitis Control Assessment Test; IQR, Interquartile range; SLIT, Subcutaneous immunotherapy.

Out of the 18 AR patients, 12 exhibited a positive response to SLIT (E-SLIT), while the remaining 6 patients showed an ineffective response to SLIT (I-SLIT). The demographics and clinical characteristics of these patients are listed in **Table 3**, with no significant differences observed in age, gender, mode of delivery, family history of allergy, feeding patterns before 6 months of age, and defecation between the two groups. However, the BMI of AR patients in the I-SLIT group was significantly lower than that in the E-SLIT group.

**Table 3:** Demographics and clinical characteristics of AR patients with effective sublingual immunotherapy (E-ELIT) and ineffective sublingual immunotherapy (I-ELIT).

Characteristics	E-SCIT	I- SCIT	*P* value
Subjects (n)	12	6	
Age (years, Mean ± SD)	8.73 ± 2.47	7.01 ± 1.24	0.134
Gender, n (%)			0.627 ^a^
Male	8 (66.7%)	3 (50.0%)	
Female	4 (33.3%)	3 (50.0%)	
BMI (kg/m2)	18.56 ± 4.72	14.91 ± 1.08	0.009
Mode of delivery, n (%)			
Vaginal	8 (66.7%)	3 (50.0%)	0.627 ^a^
Cesarean	4 (33.3%)	3 (50.0%)	
Family history of allergy, n (%)			
No	4 (33.3%)	3 (50.0%)	0.627 ^a^
Yes	8 (66.7%)	3 (50.0%)	
Feeding patterns before 6 months of age, n (%)			
Exclusively breastfeeding	7 (58.3%)	4 (66.7%)	1.000 ^a^
Mixed feeding	5 (41.7%)	2 (33.3%)	
Formula feeding	0 (0.0%)	0 (0.0%)	
Defecation, n (%)			
Regular defecation	5 (41.7%)	3 (50.0%)	1.000 ^a^
Constipation	7 (58.3%)	3 (50.0%)	
Total IgE	802.53 ± 77.20	358.17 ± 265.94	0.190
Der-f sIgE (grades)			0.567 ^a^
III	1 (8.3%)	2 (33.3%)	
IV	3 (25.0%)	1 (16.7%)	
V	5 (41.7%)	1 (16.7%)	
VI	3 (25.0%)	2 (33.3%))	
Der-p sIgE			0.762 ^a^
III	0 (0.0%)	1 (16.7%)	
IV	2 (16.7%)	1 (16.7%)	
V	6 (50.0%)	3 (50.0%)	
VI	4 (33.3%)	1 (16.7%)	

AR, Allergic rhinitis; BMI, Body Mass Index; Der-f, Dermatophagoides farina; Der-p, Dermatophagoides pteronyssinus; sIgE, specific IgE.

a Fisher’s exact test.

### 3.5 SLIT treatment altered the gut microbial composition and function of AR patients

**Figure 4:**
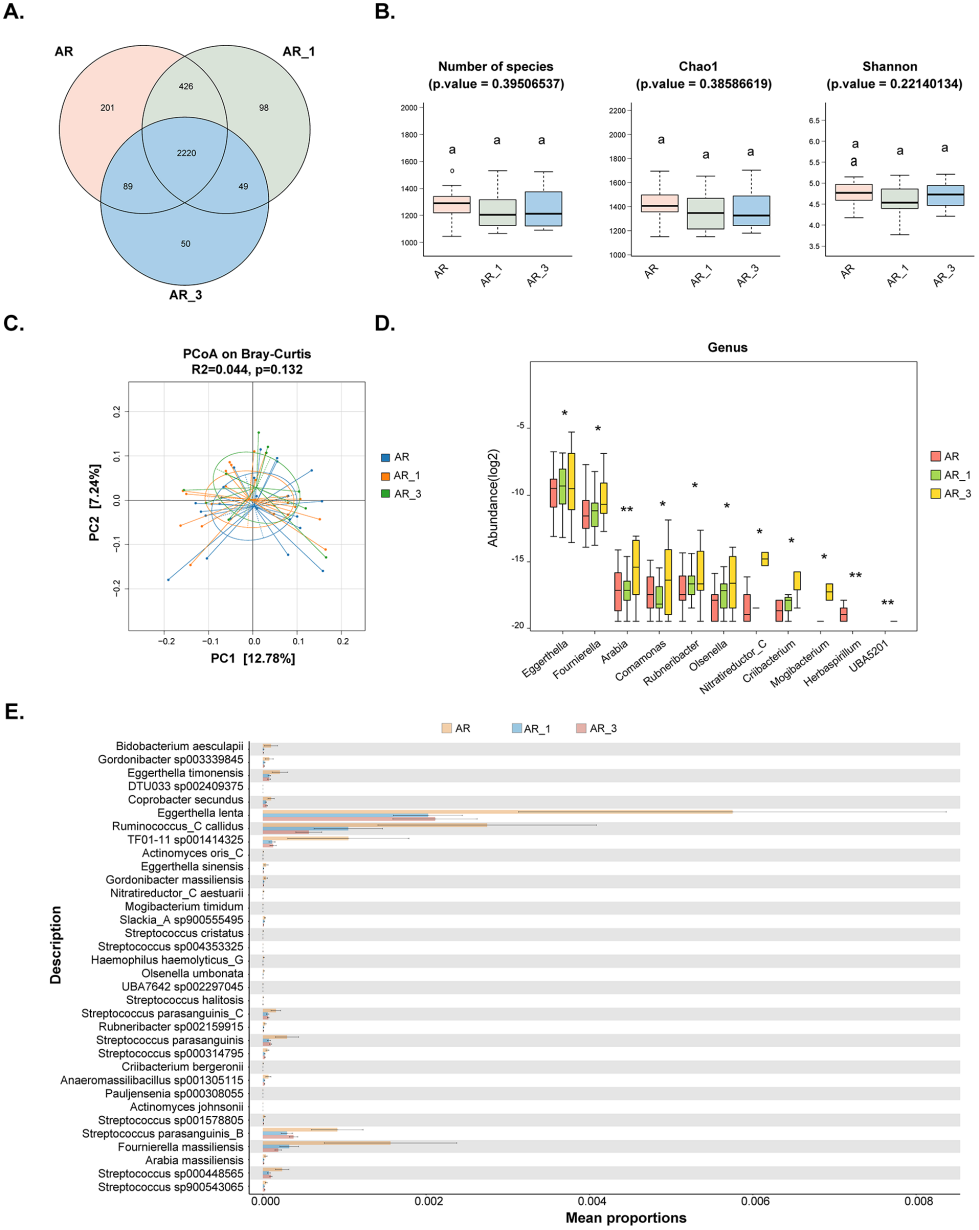
Comparisons of the gut microbiota diversity and abundance in AR patients before and at 1 and 3 months after subcutaneous immunotherapy (SLIT) treatment. **(A)** Venn diagram illustrating the overlap of observed ASVs among groups. **(B)** Alpha diversity comparison among groups, including species richness (Number of species), Chao1, and Shannon index. **(C)** Principal Coordinate Analysis (PCoA) based on Bray-Curtis distance metrics, displaying the distribution of samples along the first two principal coordinate (PC) axes. Each point represented an individual sample. **(D)** Bar plots illustrating the relative abundance of microbiota with significant differences at the genus level among the three groups. **(E)** STAMP analysis of the relative abundance of microbiota species with significant differences among the three groups. AR_1: AR patients at 1 month after treatment; AR_3: AR patients at 3 months after treatment.

Fecal samples were collected from 22 patients at 1 month and 10 patients at 3 months after SLIT treatment. Metagenomic sequencing was utilized to compare the gut microbiome of AR patients before treatment and at 1 and 3 months after treatment. The results indicated that a total of 2220 ASVs were shared among the groups, with 201 ASVs unique to the non-treatment (AR) group, 98 ASVs unique to the 1-month treatment (AR_1) group, and 50 ASVs unique to the 3-month treatment (AR_3) group (**Figure 4A**). However, neither the α-diversity nor the β-diversity of the gut microbiome changed after SLIT treatment (**Figures 4B, C**). Differential microbes were only observed at the genus and species levels, with a total of 11 genera and 34 species identified as significantly different among the groups (**Figures 4D, E**).

**Figure 5:**
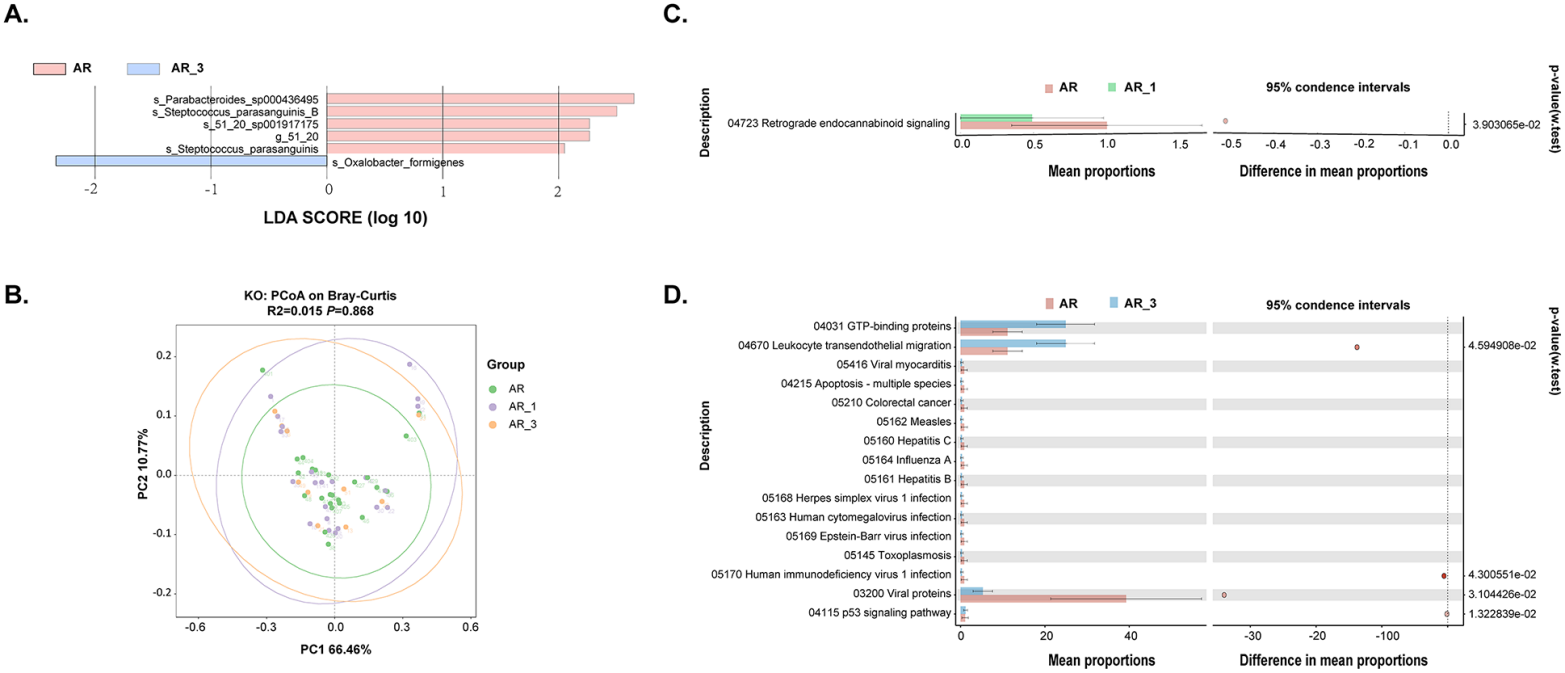
Analysis of gut microbiota composition and functionality in AR patients pre- and post- subcutaneous immunotherapy (SLIT) treatment. **(A)** Comparison of gut microbiota structures in AR patients before and 3 months after SLIT treatment, as determined by LEfSe analysis. **(B)** Principal coordinate analysis (PCoA) of KEGG orthology (KO) based on Bray-Curtis distance metrics among groups. **(C, D)** STAMP analysis revealing 1 and 16 KEGG pathways showing significant differences in abundance between AR patients before and 1 month after SLIT treatment, and AR patients before and 3 months after SLIT treatment, respectively.

We further investigated biomarkers using LEfSe among the groups, revealing that 5 microbial taxa were significantly enriched in the AR_3 group, including P*arabacteroides sp000436495*, *S. parasanguinis_B*, *51_20 sp001917175*, *51_20*, and *S. parasanguinis*, while *Oxalobacter formigenes* exhibited higher abundances in the AR group (**Figure 5A**). However, no significant difference in microbial taxa was found between the AR and AR_1 group.

Further functional analysis of the gut microbiota was conducted among AR patients before treatment and at 1 and 3 months after treatment. The dimensionality reduction PCoA revealed no significant difference in KO among the groups (**Figure 5B**). Only one signaling pathway, Retrograde endocannabinoid signaling, exhibited a significant decrease after 1-month SLIT treatment in the AR patients (**Figure 5C**). Additionally, four signaling pathways, including Leukocyte transendothelial migration, Human immunodeficiency virus 1 infection, Viral proteins, and p53 signaling pathway, showed significant differences before and after 3-month SLIT treatment in the AR patients (**Figure 5D**).

Taken together, these findings suggest that compared to 1 month, SLIT treatment for 3 months resulted in more significant changes in the microbial composition and function of AR patients.

### 3.6 The relative abundance of *S. parasanguinis_B* and *S. parasanguinis* decreased in AR patients but increased following SLIT treatment

**Figure 6:**
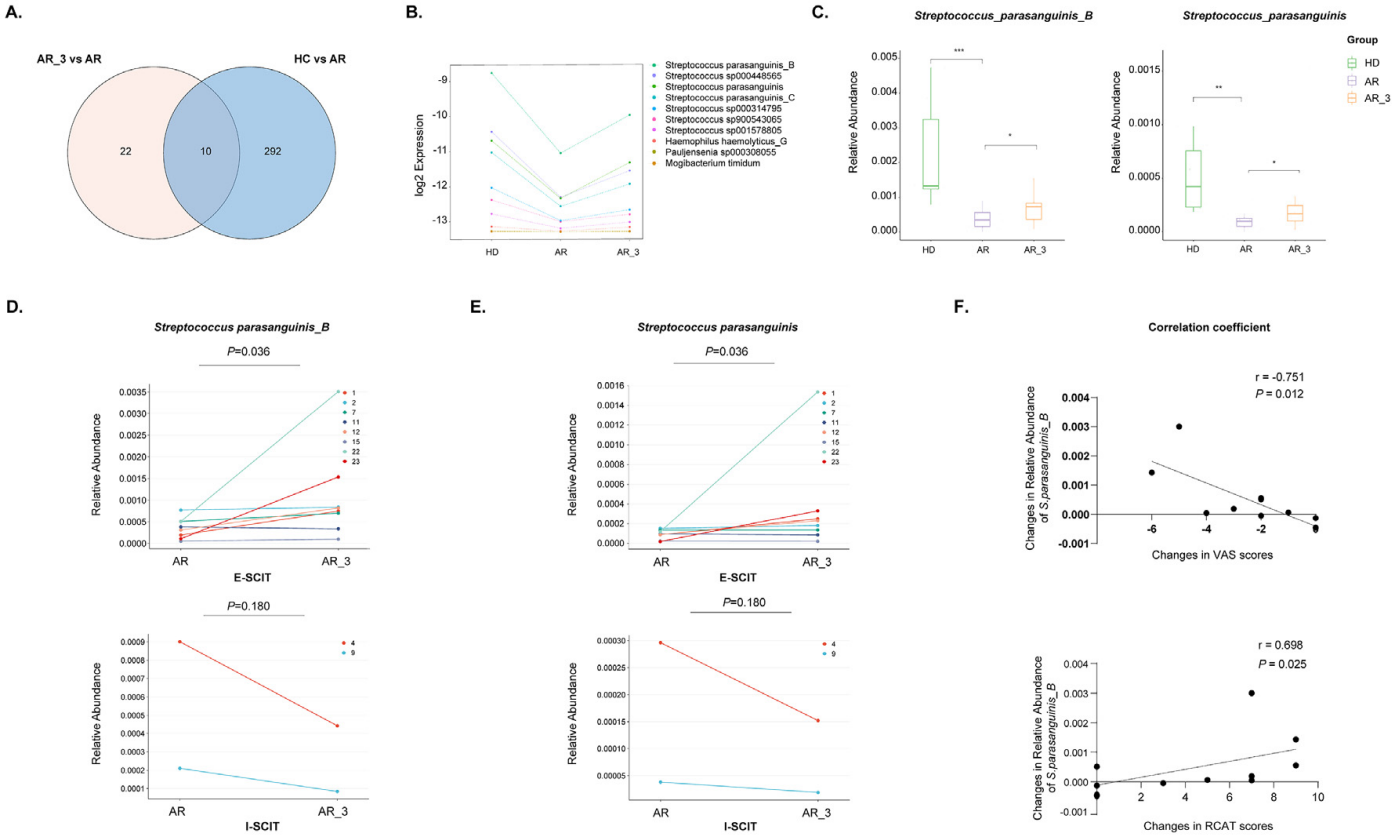
Identification of microbiota species correlated with effective SLIT treatment. **(A)** Venn diagram comparing microbiota species notably downregulated in the AR group compared to the HD group with those exhibiting significant upregulation following 3 months of SLIT treatment compared to before SLIT treatment. **(B)** The relative abundance of the 10 identified microbiota species among the groups. **(C)** The relative abundance of *S. parasanguinis_B* and *S. parasanguinis* among groups. **(D, E)** The relative abundance of *S. parasanguinis_B* and *S. parasanguinis* in each AR patient from the effective SLIT (E-SLIT) group and the ineffective SLIT (I-SLIT) group before and after treatment. **(F)** Correlation analysis showing the association between the alterations in the relative abundance of *S. parasanguinis_B* pre- and post-SLIT treatment (calculated as the abundance post-treatment subtracted by the abundance pre-treatment) and the changes in VAS and RCAT scores pre- and post-treatment (calculated as the score post-treatment subtracted by the score pre-treatment). * p < 0.05, ** p < 0.01, and *** p < 0.005.

To identify gut species influencing the efficacy of SLIT, we conducted an analysis of species notably downregulated in the AR group compared to the HD group, contrasting them with those that exhibited significant upregulation following 3 months of SLIT treatment compared to before SLIT treatment. The Venn diagram displayed a total of 10 species intersecting between the two groups (**Figure 6A**). The relative abundance of these 10 species in each group is shown in the **Figure 6B**.

Furthermore, the 10 species were intersected with those that could significantly distinguish AR group and HD group (**Figure 2B**), as well as those that could significantly distinguish AR_3 group and AR group (**Figure 5A**). Thereafter, two species, namely *S. parasanguinis_B* and *S. parasanguinis*, were identified. These species demonstrated a significant decrease in relative abundance within the AR group compared to HD group, alongside a significant increase 3 months post-SLIT treatment (**Figure 6C**).

### 3.7 The relative abundance of *S. parasanguinis_B* was positive correlated with effective SLIT treatment

To investigate the relationship between the relative abundance of *S. parasanguinis_B* and *S. parasanguinis* and the effectiveness of SLIT treatment, we analyzed the relative abundance of these two species in each AR patient from both the E-SLIT and I-SLIT groups before and 3 months after treatment. The results indicated that, except for one patient who experienced a slight decrease in the relative abundance of *S. parasanguinis_B* following SLIT treatment, all other individuals in the E-SLIT group showed an increase in both of the species after treatment. Interestingly, in the I-SLIT group, they all exhibited a decrease after treatment (**Figures 6D, E**).

Furthermore, correlation analysis was conducted to examine the relationship between the changes in the relative abundance of the aforementioned two species before and after SLIT treatment (calculated as the relative abundance of species after 3 months of treatment minus the relative abundance before treatment) and the changes in VAS score and RCAT score before and after SLIT treatment (calculated as the score after treatment minus the score before treatment). As shown in **Figure 6F**, the change of relative abundance of *S. parasanguinis_B* was negatively correlated with the changes in VAS score, but positively correlated with the changes in RCAT score before and after SLIT treatment. Taken together, these results suggested that the change of relative abundance of *S. parasanguinis_B* was positively correlated with effective SLIT treatment.

## 4 Discussion

SLIT has been demonstrated as a safe and effective treatment for AR, significantly alleviating symptoms and reducing the need for medications, thereby enhancing the overall quality of life for AR patients. However, the impact of SLIT on gut microbiota and its correlation with treatment efficacy remains unclear. Herein, we investigated difference in the gut microbiota between AR patients and HD, as well as changes in the gut microbiota of AR patients before and after SLIT treatment, and correlated these changes with the effectiveness of SLIT. This study yielded four main findings: (1) Compared to HD, AR patients exhibited altered composition and function of the gut microbiota; (2) Three months after SLIT treatment, significant alterations in the composition and function of the gut microbiota in AR patients were observed compared to pre-treatment; (3) The relative abundance of *S. parasanguinis_B* in the gut, which was significantly lower in the AR group compared to HD, exhibited a significant increase after SLIT treatment; (4) The change in the relative abundance of *S. parasanguinis_B* before and after SLIT treatment was positively associated with the effectiveness of the SLIT treatment.

Numerous studies have investigated the role of gut microbiota in the pathogenesis of AR, yet these findings have shown inconsistency (Kaczynska et al., 2022; Wan et al., 2023). Additionally, most of the previous research has relied on 16S rRNA sequencing, whereas further investigation utilizing metagenomic sequencing, which enables analysis of the gut microbial community at the species levels, is still warranted to better understand the role of gut microbiota in AR. In this study, we compared the gut microbiota of AR patients and HD through metagenomic sequencing. Our findings revealed significant alterations in both the composition and functionality of the gut microbiota in AR patients compared to HD, consistent with previous research (Zhu et al., 2020; Watts et al., 2021; Zhou et al., 2021). The variations in microbial composition were detected across different taxonomic levels, from the phylum down to the species level. The relative abundance of the Firmicutes phylum was significantly decreased in AR patients compared to that in healthy children, aligning with some previous studies (Watts et al., 2021; Zhou et al., 2021). The Firmicutes phylum can ferment dietary fiber to produce short-chain fatty acids (SCFAs), such as butyrate, propionate, and acetate (Fan and Pedersen, 2021). These SCFAs influence host metabolism and the development of allergic diseases through various mechanisms, including interacting with G protein-coupled receptors (GPCRs) expressed on intestinal enteroendocrine cells (Yang et al., 2024). Of note, several factors have been reported to be associated with the development of AR, including dietary patterns, antibiotic use, environmental exposures, and microbial exposure in early life (Ni et al., 2019; Lu et al., 2020; Liu et al., 2022b; Sahoyama et al., 2022). Especially, the gut microbiota, primarily composed of bacteria, is dynamic and shaped by the host’s dietary habits (Sahoyama et al., 2022). These microorganisms produce bioactive metabolites from dietary components, significantly impacting the host’s immune response. Dysbiosis resulting from these factors can impair the immune system’s ability to tolerate allergens, potentially increasing the risk of AR. These potential confounding factors that might influence the occurrence of AR and the efficacy of SLIT should be included in future gut microbiota studies.

The gut microbiota plays a critical role in modulating the immune response (Kaczynska et al., 2022). In AR, dysbiosis of the gut microbiota primarily disrupts the balance between Th1 and Th2 cells, promoting Th2 polarization and impairing the function of regulatory T cells (Tregs). Intestinal epithelial cells and dendritic cells facilitate interactions with gut bacteria, thereby shaping immune responses. Moreover, type 2 innate lymphoid cells (ILC2s) are also reported to be influenced by gut microbiota dysbiosis, further exacerbating AR (Kaczynska et al., 2022; Li et al., 2024). Accumulating evidence has well demonstrated that modifying the gut microbiota can suppress inflammation and alleviate AR. One of the most direct pieces of evidence is that fecal microbiota transplantation (FMT) can modify the gut microbiota in mice and alleviate inflammation in AR (Dong et al., 2024). Additionally, numerous mice and human studies have shown the efficacy of probiotics in the prevention and treatment of AR (Wang et al., 2004; Kang et al., 2020; Yan et al., 2022; Li et al., 2023). Importantly, a placebo-controlled, double-blind randomized controlled trial conducted on AR patients demonstrated that combining AIT with a symbiotic mixture containing *Bifidobacterium* spp., *Streptococcus thermophilus*, *Lactobacillus* spp., and fructooligosaccharides led to a decrease in *IL-17* gene expression by inhibiting Th17 cells compared to the group receiving only AIT or placebo (Dehnavi et al., 2019; Li et al., 2023). This result indicates that modulating gut microbiota or using specific bacterial strains as probiotics may improve the effectiveness of AIT.

However, to data, the impact of AIT on the gut microbiota of AR patients, as well as the influence of these changes on the effectiveness of AIT treatment, remains unclear. Most recently, Liu et al. have demonstrated changes in the composition and function of oral and gut microbiota in patients with atopic dermatitis undergoing AIT, and FMT from AIT-responsive patients into mice has shown to ameliorate eczema-like skin inflammation (Liu et al., 2023). In adults allergic to peanuts, AIT was also shown to augment their gut microbiota diversity (He et al., 2021). Furthermore, an elevated proportion of the salivary bacterium *Prevotella* is linked to clinical remission in pollinosis patients undergoing SLIT (Oka et al., 2021). These findings suggest that AIT could lead to changes in the gut microbiota of patients with allergic diseases, potentially impacting treatment outcomes. In the present study, we have observed, for the first time, that the gut microbiota in AR patients are altered three months after SLIT treatment, which indicated that SLIT may exert its therapeutic effects by modulating the gut microbiota of AR patients. Mechanically, SLIT may influence gut microbiota of the AR patients by enhancing the translocation of beneficial oral bacteria to the gut, thereby improving microbial composition and diversity. Additionally, the introduction of allergen under the tongue might stimulate immune responses that favor beneficial microbes, while altering the gut environment to increase the production of metabolites like short-chain fatty acids (SCFAs), which support healthy microbial communities.

According to the findings from LEfSe and random forest analysis, we identified 10 bacterial species capable of significantly distinguishing AR patients from healthy individuals, nine of which were notably reduced in AR patients. Among the species significantly reduced in AR patients, five were *Streptococcus* species, including *S. parasanguinis_B*, *S. parasanguinis_C*, *S. parasanguinis*, *S. sp000448565*, and *S. rubneri*. Notably, *S. parasanguinis_B* and *S. parasanguinis* also exhibited significant increases in AR patients three months after SLIT treatment and served as biomarkers to distinguish AR patients before and after SLIT treatment. Additionally, apart from one patient who experienced a slight decrease in the relative abundance of *S. parasanguinis_B* following SLIT treatment, all other individuals in the E-SLIT group showed an increase in *S. parasanguinis_B* and *S. parasanguinis* after treatment. Conversely, in the I-SLIT group, they both exhibited a decrease after treatment in each individual. Furthermore, it was found that the change in the relative abundance of *S. parasanguinis_B* was positively correlated with the efficacy of SLIT treatment. These results suggest SLIT may partially exert its therapeutic effects by modulating the abundance of *S. parasanguinis_B*.

*S. parasanguinis*, which is a commensal Gram-positive bacterium, serves as a primary colonizer of the human oral cavity and various body sites (Franzosa et al., 2014). It plays a critical role in dental plaque formation by regulating microbiome metabolism through hydrogen peroxide production, which interacts with nitrite to generate antimicrobial reactive nitrogen species (RNS). This process favors commensal bacteria and promotes protective metabolites like para-aminobenzoic acid (PABA). Besides, *S. parasanguinis* demonstrates resilience to nitrosative stress through enhanced nucleotide turnover and increased aromatic compounds from the Shikimate pathway, supporting its growth and inhibiting pathogen virulence factors, thereby maintaining oral health and microbial balance (Herrero et al., 2016; Chen et al., 2019; Huffines and Scoffield, 2020; Huffines et al., 2022). Additionally, *S. parasanguinis* is reported to be the dominant pioneer colonizer of the human infant intestine in the first days of life, as well as one of the predominant bacterial species in the small intestine of adults (Songjinda et al., 2005; van den Bogert et al., 2013). As a butyrate-producing species, it exerts immunomodulatory effects and promotes the differentiation of regulatory T cells (Tregs). It has been shown to protect against cardiovascular diseases by producing butyrate and ketone bodies, which modulate immune responses (Chen et al., 2023). Additionally, it has been negatively correlated with fecal citrulline levels in patients with unstable angina, which is a substrate for arginine recycling essential for nitric oxide synthesis (Liu et al., 2022a). It also possesses anti-inflammatory properties, which may contribute to the inhibition of AR development. Furthermore, previous studies indicated that oral bacteria can translocate to the gut, with *S. parasanguinis* being one of the most abundant species involved in this process (Kageyama et al., 2023). SLIT, which involves placing allergen extracts under the patient’s tongue, may facilitate the translocation of *S. parasanguinis* from the oral cavity to the gut. This translocation could further enhance the effectiveness of SLIT in treating AR. However, the impact and underlying mechanisms of *S. parasanguinis* on AR development and the efficacy of SLIT still require further investigation.

Our study still has some limitations. Firstly, the impact of *S. parasanguinis_B* on the efficacy of SLIT treatment has not been validated through animal experiments, resulting in a lack of direct evidence confirming its effectiveness. Secondly, the mechanisms by which *S. parasanguinis_B* contribute to SLIT treatment for AR were not explored in this study. These concerns will be fully investigated in our future work.

Taken together, we found that SLIT treatment can alter the composition of the gut microbiota in AR patients, and *S. parasanguinis_B* may be associated with the effectiveness of SLIT treatment. Our study provides new insights into the mechanisms of SLIT treatment for AR and offers the possibility of using specific bacterial strains as biomarkers for the effectiveness of SLIT treatment, as well as regulating those specific bacterial strains to enhance the effectiveness of SLIT.

## Data Availability

The data presented in the study are deposited in the China National Center for Bioinformation, Genome Sequence Archive (GSA) for human repository, accession number CRA020176.

## Supplementary material

Supplementary Figure 1 Flowchart of the present study.

Supplementary Figure 2 Comparisons of the gut microbiota diversity between AR patients and healthy donors (HD). **(A)** Comparison of Simpson index between groups. **(B)** Principal coordinate analysis based on Bray-Curtis distance metrics was shown along the first two principal coordinate (PC) axes. Each point represented a single sample.
