# Supplementary figures and images for: Allergen-specific sublingual immunotherapy altered gut microbiota in patients with allergic rhinitis
Source: Front Cell Infect Microbiol. 2024 Nov 8;14:1454333. doi: 10.3389/fcimb.2024.1454333 (PMC11626388; doi:10.3389/fcimb.2024.1454333)

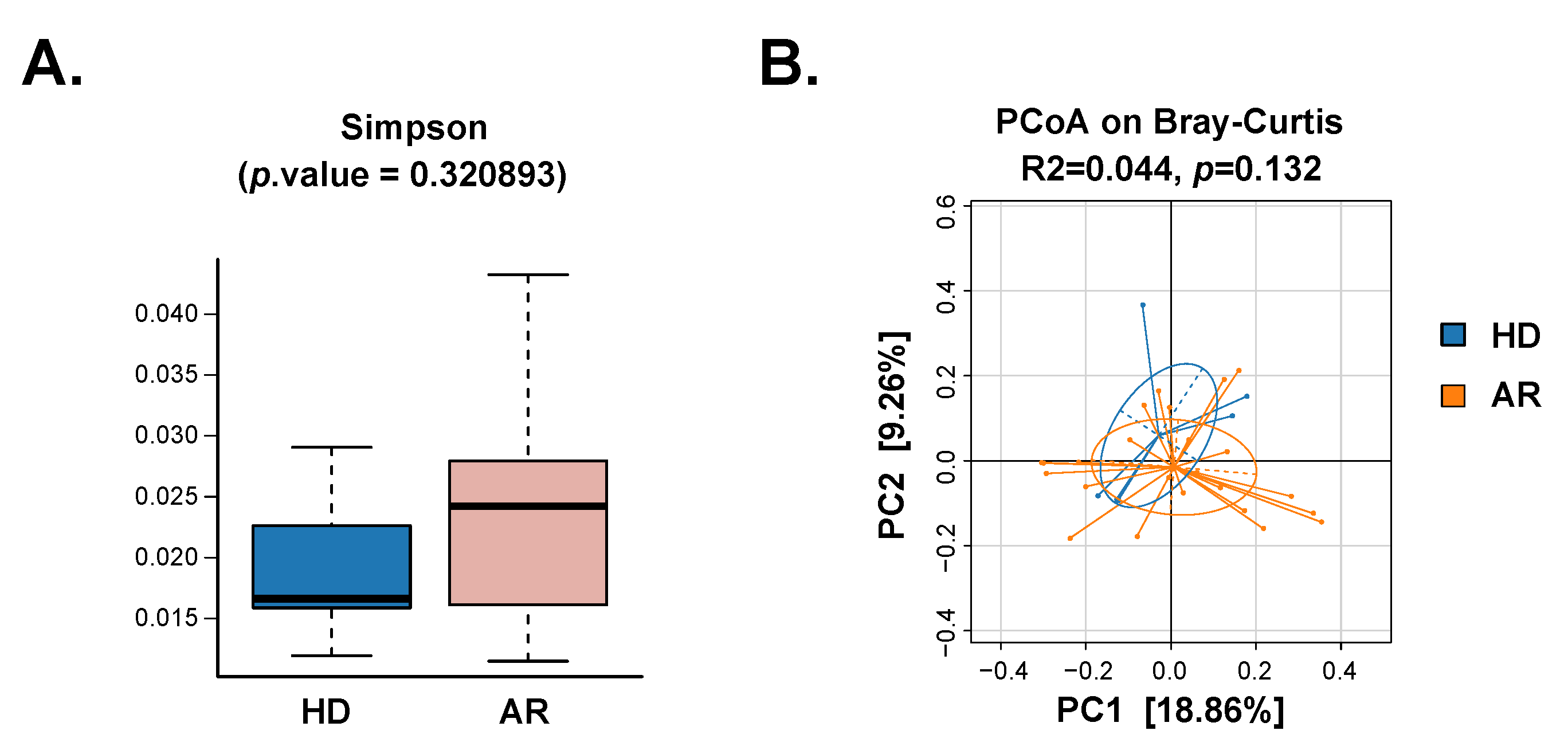

Supplement: Supplementary Figure 2 — Comparisons of the gut microbiota diversity between AR patients and healthy donors (HD). (A) Comparison of Simpson index between groups. (B) Principal coordinate analysis based on Bray-Curtis distance metrics was shown along the first two principal coordinate (PC) axes. Each point represented a single sample. [file Image2.tiff]
