# Supplementary material for: Allergen-specific sublingual immunotherapy altered gut microbiota in patients with allergic rhinitis
Source: Front Cell Infect Microbiol. 2024 Nov 8;14:1454333. doi: 10.3389/fcimb.2024.1454333 (PMC11626388; doi:10.3389/fcimb.2024.1454333)
Supplement: Supplementary file 5 [file Table3.docx]

**Table S3. VAS score and RCAT score pre- and post-SLIT in AR patients.**

| Pts | VAS score | | RCAT score | | Nasal  congestion | | | Sneezing | | | Watery eyes | | | Sleep problems caused by rhinitis | | | | Activity avoidance | | Rhinitis symptom control | |
| --- | --- | --- | --- | --- | --- | --- | --- | --- | --- | --- | --- | --- | --- | --- | --- | --- | --- | --- | --- | --- | --- |
| **Pts** | Pre | Post | Pre | Post | | Pre | Post | | Pre | Post | | Pre | Post | | Pre | Post | Pre | | Post | Pre | Post |
| **1** | 9 | 7 | 12 | 21 | | 2 | 3 | | 2 | 3 | | 2 | 4 | | 2 | 3 | 3 | | 5 | 1 | 3 |
| **2** | 8 | 7 | 18 | 23 | | 5 | 4 | | 4 | 5 | | 2 | 3 | | 1 | 3 | 3 | | 5 | 3 | 3 |
| **4** | 6 | 6 | 23 | 23 | | 3 | 3 | | 5 | 5 | | 4 | 4 | | 3 | 3 | 5 | | 5 | 3 | 3 |
| **5** | 6 | 3 | 24 | 29 | | 2 | 4 | | 4 | 5 | | 5 | 5 | | 3 | 5 | 5 | | 5 | 5 | 5 |
| **6** | 8 | 8 | 21 | 21 | | 2 | 2 | | 5 | 5 | | 4 | 4 | | 2 | 2 | 5 | | 5 | 3 | 3 |
| **7** | 8 | 5 | 13 | 20 | | 1 | 3 | | 2 | 4 | | 1 | 1 | | 3 | 3 | 4 | | 5 | 2 | 4 |
| **9** | 6 | 6 | 27 | 27 | | 3 | 3 | | 5 | 5 | | 5 | 5 | | 5 | 5 | 5 | | 5 | 4 | 4 |
| **10** | 7 | 5 | 24 | 25 | | 3 | 4 | | 3 | 3 | | 5 | 5 | | 4 | 4 | 5 | | 5 | 4 | 4 |
| **11** | 4 | 2 | 17 | 20 | | 2 | 3 | | 2 | 3 | | 1 | 1 | | 4 | 4 | 5 | | 5 | 3 | 4 |
| **12** | 8 | 6 | 26 | 26 | | 2 | 2 | | 5 | 5 | | 5 | 5 | | 5 | 5 | 5 | | 5 | 4 | 4 |
| **13** | 2 | 2 | 26 | 26 | | 5 | 5 | | 3 | 3 | | 5 | 5 | | 5 | 5 | 4 | | 4 | 4 | 4 |
| **14** | 6 | 2 | 21 | 26 | | 2 | 4 | | 3 | 4 | | 4 | 4 | | 4 | 5 | 5 | | 5 | 3 | 4 |
| **15** | 6 | 2 | 20 | 27 | | 3 | 5 | | 1 | 3 | | 3 | 5 | | 5 | 5 | 5 | | 5 | 3 | 4 |
| **17** | 8 | 3 | 15 | 27 | | 2 | 3 | | 2 | 5 | | 2 | 5 | | 3 | 5 | 4 | | 5 | 2 | 4 |
| **20** | 5 | 5 | 23 | 23 | | 4 | 4 | | 3 | 3 | | 3 | 3 | | 5 | 5 | 5 | | 5 | 3 | 3 |
| **21** | 7 | 7 | 23 | 23 | | 5 | 5 | | 4 | 4 | | 5 | 5 | | 4 | 4 | 3 | | 3 | 2 | 2 |
| **22** | 7 | 2 | 19 | 26 | | 3 | 4 | | 3 | 3 | | 3 | 5 | | 2 | 5 | 5 | | 5 | 3 | 4 |
| **23** | 8 | 2 | 21 | 30 | | 2 | 5 | | 2 | 5 | | 5 | 5 | | 4 | 5 | 5 | | 5 | 3 | 5 |
